# Effect of Guest Atom Composition on the Structural and Vibrational Properties of the Type II Clathrate-Based Materials A_x_Si_136_, A_x_Ge_136_ and A_x_Sn_136_ (A = Na, K, Rb, Cs; 0 ≤ *x* ≤ 24)

**DOI:** 10.3390/ma9080691

**Published:** 2016-08-11

**Authors:** Dong Xue, Charles W. Myles, Craig Higgins

**Affiliations:** Department of Physics, Texas Tech University, Lubbock, TX 79409-1051, USA; Dong.Xue@ttu.edu (D.X.); Craig.Higgins@gmail.com (C.H.)

**Keywords:** clathrates, type II-structure, guests, alkali atoms, Group 14, first principles theory, VASP, phonon, elastic constants, 63.20dk, 63.20Pw, 61.50Ah, 61.66Fn

## Abstract

Type II clathrates are interesting due to their potential thermoelectric applications. Powdered X-ray diffraction (XRD) data and density functional calculations for Na_x_Si_136_ found a lattice contraction as x increases for 0 < x < 8 and an expansion as x increases for x > 8. This is explained by XRD data that shows that as x increases, the Si_28_ cages are filled first for x < 8 and the Si_20_ cages are then filled for x > 8. Motivated by this work, here we report the results of first-principles calculations of the structural and vibrational properties of the Type II clathrate compounds A_x_Si_136_, A_x_Ge_136_, and A_x_Sn_136_. We present results for the variation of the lattice constants, bulk moduli, and other structural parameters with x. These are contrasted for the Si, Ge, and Sn compounds and for guests A = Na, K, Rb, and Cs. We also present calculated results of phonon dispersion relations for Na_4_Si_136_, Na_4_Ge_136_, and Na_4_Sn_136_ and we compare these for the three materials. Finally, we present calculated results for the elastic constants in Na_x_Si_136_, Na_x_Ge_136_, and Na_x_Sn_136_ for *x* = 4 and 8. These are compared for the three hosts, as well as for the two compositions.

## 1. Introduction

Compounds which have th Type-II, Group 14 clathrate lattice structure have generated considerable interest in recent years and several experimental and theoretical studies of these materials have been carried out. A primary reason for why such materials are interesting is that they have very good electrical transport properties while simultaneously having glass-like thermal transport properties. Materials with both good electrical transport properties and poor thermal transport properties are not very common. However, it is well-known that materials which simultaneously satisfy both criteria have potential applications in thermoelectrics.

There have been several investigations of both “guest-free” clathrates, formed by face-shared polyhedra cages of Group 14 atoms [1,2,3,4,5,6,7,8,9] and of the compounds formed when alkali metal impurities (guests) are put inside the lattice cages. It has been shown that the presence of these guest atoms can improve the material thermoelectric (TE) performance. This better TE performance is qualitatively attributable to an increase in the thermoelectric figure-of-merit (ZT) [10,11] of the guest-containing clathrates in comparison with the guest-free materials. From a theoretical viewpoint, achieving a large ZT requires that the total thermal conductivity κ (including the lattice and electronic contributions) be minimized. Previous studies of the guest-containing Type II clathrate compounds [12,13] have shown that their low κ is due to the presence of low lying guest atom-induced vibrational (“rattling”) modes, which can scatter from the heat carrying acoustic modes of the host lattice, thus suppressing the phonon contribution to the material heat conduction.

Recently, binary Type II clathrate based compounds with cubic space group symmetry $Pm\bar{3}n$ have attracted considerable attention [14,15,16,17,18,19]. The 136 atom unit cell of these materials contains two different sized cages: dodecahedra (20-atom cages) and hexakaidecahedra (28-atom cages). As was just mentioned, encapsulated guest atom “rattlers” (such as Na, K, Rb, Cs) vibrating inside the large and small cages can have significant effects on the low-lying vibrational modes of the material [20,21], which can help to minimize the lattice thermal conductivity. In fact, such materials can be shown to satisfy the “Phonon Glass Electron Crystal (PGEC)” criteria for thermoelectrics proposed originally by Slack [22].

In this paper, we report the results of a first-principles, density functional based, computational and theoretical study of the structural and vibrational properties of the Type II clathrate-based compounds A_x_M_136_ (A = Na, K, Rb, Cs; M = Si, Ge, Sn; 0 ≤ *x* ≤ 24). We compare some of the results of our calculations with experimental data for some of the framework-substituted ternary clathrate compounds such as Rb_8_Ga_8_Si_128_, Cs_8_Ga_8_Si_128_, Na_16_Cs_8_Si_136_, and Na_16_Cs_8_Ge_136_ [20,21,23]. In addition, we present results for the dependence of the lattice constants and other structural properties in A_x_M_136_ (A = Na, K, Rb, Cs; M = Si, Ge, Sn) on guest composition x. For Na_x_Si_136_, we find that our calculated lattice constant as a function of x correlates well with extensive, detailed powdered X-ray diffraction (p-XRD) measurements [14]. We note that reference [14] is accompanied by an on-line Supplemental Information document, which contains many details of the XRD experiments and analyses. An analysis of this p-XRD data has shown that, as x increases from *x* = 0, the Na guests in Na_x_Si_136_ preferentially fill the 28-atom cages until each of the 8 such cages in the unit cell are full. As x increases beyond *x* = 8, the smaller 20-atom cages are then filled with Na guests.

Experimental data on the vibrational properties of Na_x_Si_136_ (*x* = 3, 24, 25) confirms the presence of low-frequency vibrational, rattling modes due to the Na guest atoms. Among the experimental techniques which have been used are temperature-dependent single-crystal XRD (which measures mean square atomic displacement amplitudes U_eq_), temperature-dependent heat capacity (C*_p_*) studies, and inelastic neutron scattering (INS) studies [15]. The results of our first principles calculations show reasonable agreement with the experimental data when comparing our calculated vibrational energy of 6.2 meV (at *x* = 4) for Na in the Si_28_ cages with the experimentally determined Na atom rattler frequency of 6.5 meV in Na_3_Si_136_. An intriguing result of our calculations is that we find that Na becomes more strongly bound with respect to the M_28_ (M = Si, Ge, Sn) cage, as atomic weight of the host atom changes from 28 for Si, to 72.6 for Ge, and 118.7 for Sn. This result is contrary to a previous calculation of the rattling frequency of Cs guests when the host changes from Si to Ge to Sn. In that case, it was found that the effective force constant of Cs in the Sn_28_ cages is substantially reduced in comparison with its values for Cs in the Si_28_ cages and in the Ge_28_ cages [20]. On the other hand, we find that the anisotropic velocities of the heat-carrying acoustic phonons in Na_x_Sn_136_ (*x* = 4, 8) are significantly decreased in comparison to those in Na_x_Si_136_.

## 2. Computational Approach

Our first-principles calculations are based on the Local Density Approximation (LDA) to the Density Functional Theory (DFT). For most of our calculations, we have used the Vienna ab-initio Simulation Package (VASP) [24,25,26,27] and we have employed the Ceperley-Alder exchange-correlation potential along with pseudo-potentials obtained using the projector augmented wave method (PAW). Details of similar LDA-based calculations for the clathrate systems Sn_136_, Sn_46_, Rb_8_Na_16_Si_136_, Rb_8_K_16_Si_136_, as well as others are described in Ref. [7,8,28,29]. We note that there are several PAW versions of the pseudo-potential for the Rb atom. For the case of the Rb guests in the Type II clathrates considered in this study, we have used the one which treats the 3s and 3p states as valence states, rather than as core states, as is done for the Rb ultra-soft pseudo-potentials. In this sense, results obtained with the PAW pseudo-potential are expected to be more accurate [30,31].

In our calculations, the structural and vibrational properties of the A_x_M_136_ materials are calculated after the optimized geometry of each compound has been fully determined. This optimization is done by means of a conjugate gradient method, which relaxes the internal coordinates of the atoms confined in a fixed volume of the face centered cubic (FCC) unit cell. We especially point out that all of the guest atoms during these optimization processes are allowed to move freely. That is, the guest atoms are not fixed in position but are allowed to move to their own equilibrium positions. The process for the relaxation and determination of the optimized structure must be repeated many times until a global minimum total energy is achieved. Next, several pairs of calculated results for the LDA total energy vs. volume (E, V) are fitted to the 3rd order Birch-Murnaghan equation of state (EOS) [32]. This fitting procedure yields the equilibrium energy E_0_, the equilibrium volume V_0_, the bulk modulus B, and the pressure derivative of the bulk modulus, B′ = (dB/dP) at absolute zero temperature. We have used a 4 × 4 × 4 Monkhorst-Pack *k*-point grid [33] to perform the relaxation and to find the equilibrium geometry. During these calculations, the total energy convergence criterion was adjusted to 10^−7^ eV. Once the lattice parameter for a given material has been determined, the VASP code can readily perform calculations of other structural parameters as well as of the vibrational modes. In addition, the Fermi level, the electronic band structure, and the “pseudogap” for the optimized geometry can also be determined. In this paper, we focus on the results of such calculations for the structural and vibrational properties and we defer a discussion of these electronic properties to a future paper.

The procedure for calculating the vibrational properties of a material A_x_M_136_ consists of two steps. First, we obtain the dynamical matrix D(***q***), by moving each atom in the unit cell by a small finite displacement (U_0_ = 0.02 Å). The D(***q***) constructed in this manner not only corresponds to the dynamical matrix at the gamma (Γ) point [q = (0,0,0)], but D(***q***) also can be calculated for non-zero q if it is assumed that the matrix elements of D(***q***) vanish for atoms separated by a distance greater than the third nearest neighbor distance [3]. Using this approximation for calculating D(***q***), the force constant matrix is calculated with a 2 × 2 × 2 *k*-point grid using the *k*-points along certain high symmetry directions in the Brillouin zone. Second, the diagonalization of D(***q***) allows us to find the vibrational eigenvalues (squared frequencies) and eigenvectors.

## 3. Results

In order to study the effect of the guest atom species and the concentration x on the structural and the vibrational properties of the A_x_M_136_ Type II clathrate-based materials, we have calculated the x dependencies of the lattice constants, the bulk modulii, the Birch-Murnaghan parameters, the elastic constants C_44_ and C_11_, the effective force constants, and the low-lying guest-associated vibrational modes for such materials. We find that our calculated lattice constant as a function of x correlates well with the powdered X-ray diffraction (p-XRD) data for Na_x_Si_136_ [14]. (See also the extensive on-line supporting information document which accompanies reference [14]. There, many details of the Na_x_Si_136_ materials studied of the XRD data are presented and discussed.)

A previous analysis of this p-XRD data has shown that, as x increases from *x* = 0, the Na guests preferentially fill the 28-atom cages until all eight such cages in the unit cell are full. As x increases beyond *x* = 8, the smaller 20-atom cages are then filled with Na guests. Furthermore, previous LDA calculations for Na_x_Si_136_ of the lattice contraction for x < 8 and expansion of Na_x_Si_136_ for x > 8 have been shown to compare well with the XRD data. Moreover, a recent experimental study of Na-doped type II Si clathrates synthesized from NaSi powder also confirms lattice framework contraction as only large cages are partially or entirely occupied by Na atoms [34]. Although the detailed, microscopic physics mechanism governing the lattice contraction of Na_x_Si_136_ as Na is encapsulated into the Si_28_ cages remains unclear, we speculate that the volume difference between the Na guest atoms and the Si framework cage is the likely cause of such an unusual structural change upon Na filling. In addition, guest atom-framework atom bonding forces, which are weak and attractive, might also contribute to an explanation of the unit cell contraction for *x* < 8.

Our first-principles calculations of the structural properties of A_x_M_136_ have been mainly focused on two related problems. The first of these is to obtain an understanding of how various alkali guest atoms affect the behavior of the lattice constant and other structural properties of the Type II Si clathrate host. The second is to obtain an understanding of how the Na guests affect the lattice properties if the host is changed from Si to Ge to Sn. For A_x_Si_136_, our predicted minimum lattice constant as a function of composition x is 14.558 Å for Na_8_Si_136_, 14.564 Å for K_8_Si_136_, 14.572 Å for Rb_8_Si_136_, and 14.582 Å for Cs_8_Si_136_. Our estimated lattice constant for Na_8_Si_136_ is slightly smaller than the p-XRD determined value of 14.6423 Å [17].

**Figure 1:**
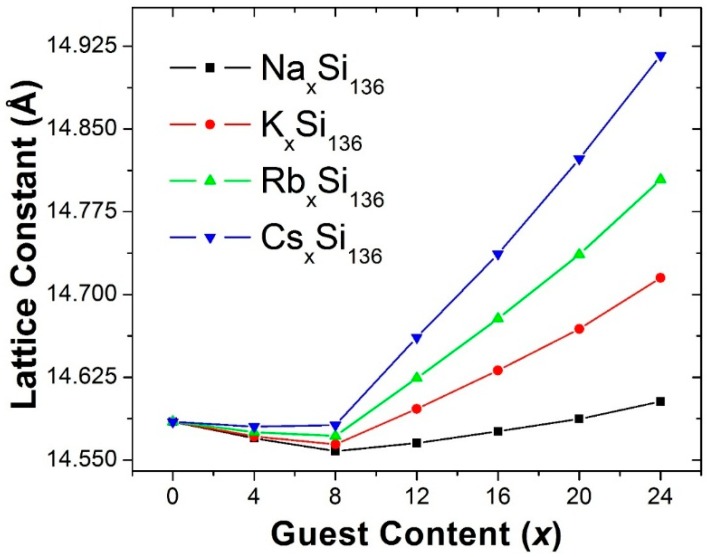
Predicted x Dependence of the Lattice Constants for the Type II Clathrate-Based Compounds. A_x_Si_136_ (A = Na, K, Rb, Cs; 0 ≤ *x* ≤ 24).

Figure 1 shows our predicted lattice constant for the four clathrate compounds A_x_Si_136_ (A = Na, K, Rb, or Cs). For Na_x_Si_136_, the large difference between the guest atom size and the cage volume for Na in the Si_28_ cages causes the unusual lattice response to filling the cages, as discussed above. That is, for this case, our predicted lattice constant decreases with x for x < 8 and increases with x for x > 8. Also, as can be seen in Figure 1, our calculations predict that for A_x_Si_128_, the incorporation of the alkali atom guests A = K, Rb, and Cs into Si_136_ in each case also results in small decreases of the lattice constant as a function of x for x < 8. These results also predict that this decrease should be less as the guest atom is changed from Na to K to Rb to Cs. In fact, for Cs_x_Si_136_, the predicted lattice constant is almost a constant for x < 8. From Figure 1, in each case it can also be seen that for x > 8, our calculated lattice constant increases as a function of x. Furthermore, the slope of the lattice constant as a function of x is predicted to become larger as the guest atom is changed from Na to K to Rb to Cs. 

Since our predicted x dependences of the lattice constants on guest composition x in Figure 1 are for all x values (0 < x < 24) and therefore are for the alkali atom guests inside both the large, 28 atom cages and the small, 20 atom cages, it is interesting to briefly consider how the size of the clathrate cage in comparison with the guest atom “size” can affect how “tightly” or “loosely” an alkali atom guest can fit inside the Si, Ge, and Sn host clathrate cages. In Ref. [20] a simple model was introduced to help to understand this. Cs was chosen as an example in Ref. [20] because it has the largest ionic radius of the alkali atoms. Since Si cages are the smallest among the Group 14 clathrates, it is useful to briefly discuss the relative sizes of the Si cages and Cs guests within the model of reference [20]. That is, considering the largest alkali guest atom (Cs) inside the smallest clathrate cages (Si) should provide insight into how easily (or not) the alkali guests will fit into the clathrate cages. 

Our LDA-calculated result for the covalent radius of silicon in the Sn_28_ cages is *r*_Si_ = 1.17 Å. An estimate of the ionic radius of Cs is *r*_Cs_ = 1.69 Å. Within the model of reference [20], the size of a Si_28_ cage is determined by the distance *r*_cage_(Cs-Si) between a Si atom in the cage structure and a Cs guest atom inside the cage. Our LDA result for this distance in the Si_28_ cages is *r*_cage_(Cs-Si) = 3.93 Å. As discussed in reference [20], a useful measure of how easily (or not) a Cs guest can be accommodated inside a Si_28_ cage is the “excess” radius which is defined as Δr ≡ *r*_cage_(Cs-Si) − (*r*_Si_ + *r*_Cs_). Using the numerical values just discussed for each of the distances that determine Δr gives an estimate for the excess radius of Δr = 1.07 Å for Cs. Therefore, the Cs guests should be relatively easily accommodated inside the large Si_28_ cages. Further, there clearly is enough room in these cages to allow for the Cs guests to undergo large amplitude vibrations. 

Similarly, our LDA calculated results for the covalent radius of silicon in the small Si_20_ cages is *r*_Si_ = 1.18 Å, and for the distance between a Si and a Cs in the same cages is *r*_cage_(Cs-Si) = 3.26 Å. This clearly shows that, as expected, the Si_20_ cages are significantly smaller than the Si_28_ cages. Applying the model of Ref. [20] to Cs in the Si_20_ cages gives an estimate of the “excess” radius for those cages as Δr ≡ *r*_cage_(Cs-Si) − (*r*_Si_ + *r*_Cs_) = 0.39 Å. That is, the Cs guests clearly have very little room inside the Si_20_ cages. Based on this analysis, the Cs atom guests should be able to be accommodated inside the Si_20_ cages. However, a Cs atom in a Si_20_ cage clearly will not have much “excess” volume to move around in. We further note that this simple analysis considers only the relative sizes of the Si cages and the Cs guest atoms. There may be other factors, such as the complicated chemistry, to consider when trying to synthesize a Cs_x_Si_136_ sample with Cs in both the large and the small cages. 

**Figure 2:**
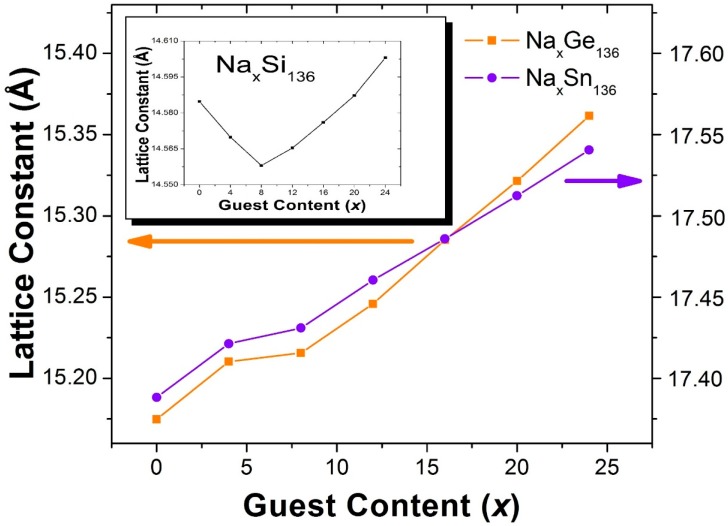
Predicted x Dependence of the Lattice Constants for Type II Clathrate-Based Compounds Na_x_M_136_ (M = Si, Ge, Sn; 0 ≤ *x* ≤ 24).

Figure 2 shows the results of our calculations of the x dependence of the lattice constants for Na_x_M_136_ for M = Si, Ge, and Sn. Unlike the structural trends we have found in A_x_Si_136_, our calculations for Na_x_M_136_ predict that there should be no lattice contraction or minimum in the lattice constant at *x* = 8 when Si is replaced by Ge or Sn. In this case, for x > 8, our calculated lattice parameters are almost linear functions of the guest concentration *x*. That is, in contrast to the Na_x_Si_136_ case, we predict that the volume difference between the encapsulated Na guest atoms and host framework cages does not lead to an appreciable lattice contraction for x < 8, and for either Na_x_Ge_136_ or Na_x_Sn_136_.

Table 1 shows our LDA calculated results for some of the equilibrium lattice structural parameters of the clathrates Na_8_Si_136_, K_8_Si_136_, Rb_8_Si_136_, Cs_8_Si_136_, Na_8_Ge_136_, and Na_8_Sn_136_. Our results for the lattice constant a_0_, the bulk modulus B, the pressure derivative of bulk modulus B′ = dB/dP, the minimum binding energy per atom E_0_, and the equilibrium volume per atom V_0_ are shown. Our results for the bulk moduli of the binary Type II Si clathrate-based materials Na_8_Si_136_, K_8_Si_136_, Rb_8_Si_136_, and Cs_8_Si_136_ predict that they should be softer than the “guest-free” Type II material Si_136_ (90 GPa) [35]. Similarly, our results predict that the Type II Ge clathrate-based material Na_8_Ge_136_ has a lower bulk modulus than the measured value for pristine Ge_136_ (61.9 GPa) [36].

**Figure 3:**
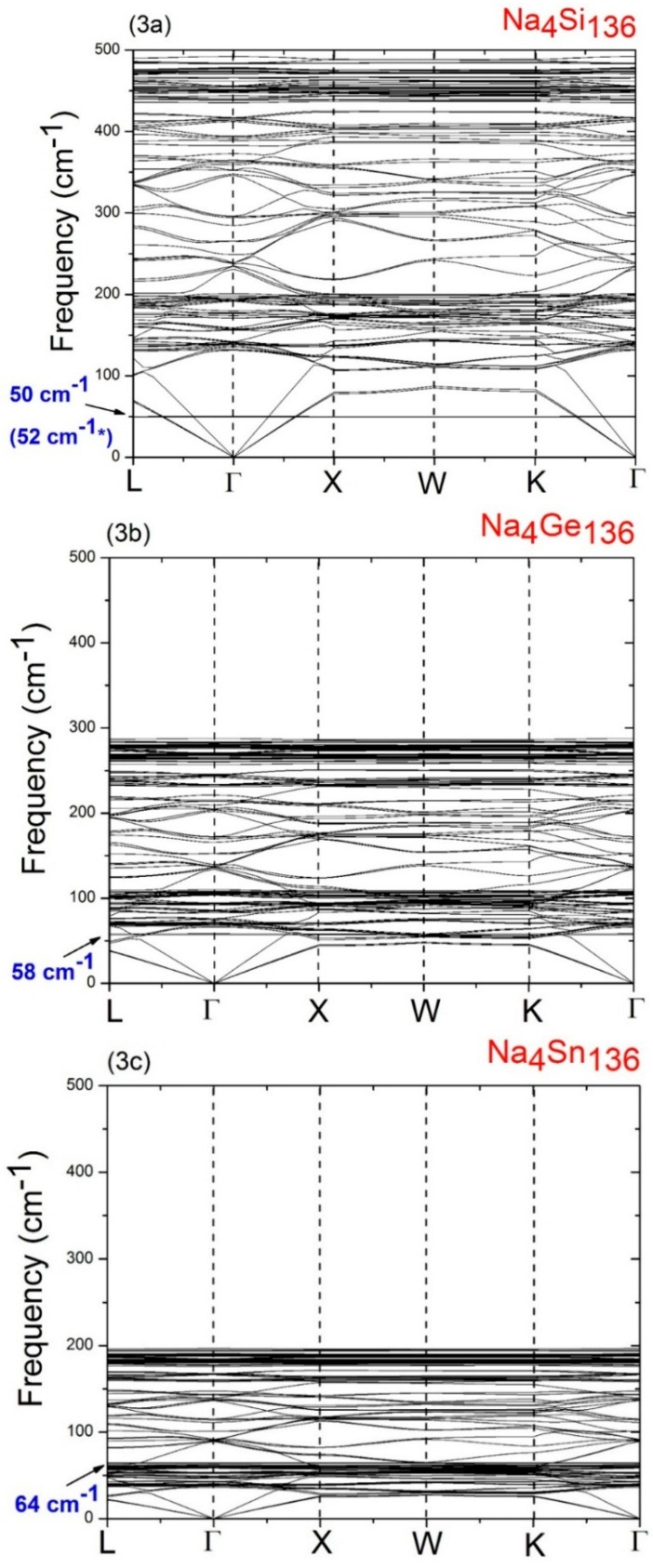
Calculated phonon dispersion curves for some of the Type II clathrate materials with four Na guests in the large, 28 atom cages. The predicted vibrational modes for Na_4_Si_136_, Na_4_Ge_136_, and Na_4_Sn_136_ are shown in Figure 3**a**–**c**, respectively. The number in parentheses is for the Na guest associated modes in Na_4_Si_136_, and was obtained from inelastic neutron scattering (INS) experiments [15].

In order to understand the behavior of the Na guests vibrating inside the 28 atom framework cages (M_28_) in the Type II clathrate materials, we have calculated the phonon dispersion relations for Na_4_Si_136_, Na_4_Ge_136_, and Na_4_Sn_136_. The results of these calculations are summarized in Figure 3a–c, respectively. As is to be expected based on the relative mass sizes of the Si, Ge, and Sn atoms, a comparison of Figure 3a–c shows clearly that the maximum host material optical frequency decreases as the material changes from Na_4_Si_136_ to Na_4_Ge_136_ to Na_4_Sn_136_. Specifically, we find that the highest optical frequencies occur at about 492 cm^−1^ in Na_4_Si_136_, at about 287 cm^−1^ in Na_4_Ge_136_, and at about 197 cm^−1^ in Na_4_Sn_136_. As is discussed in a previous study [37], such high frequency optical vibrations are due to the bond-stretching modes. However, at the same time, the occupancy of the framework antibonding states for the different species (Si, Ge, Sn) also affects the Si-Si, Ge–Ge, and Sn–Sn bond order. The results in Figure 3a–c, also predict that the maximum host acoustic mode frequency lies below about 127 cm^−1^ in Na_4_Si_136_, below about 70 cm^−1^ in Na_4_Ge_136_, and below about 48 cm^−1^ in Na_4_Sn_136_.

A detailed analysis of the vibrational modes in Na_4_Si_136_, Na_4_Ge_136_, and Na_4_Sn_136_ shows that the LDA-calculated low lying isotropic vibrational (“rattling”) frequencies of the Na guests are about 50 cm^−1^ for Na_4_Si_136_, 58 cm^−1^ for Na_4_Ge_136_, and 64 cm^−1^ for Na_4_Sn_136_. In order to understand the variation of these low frequency Na guest-associated modes as the host material changes from Si_136_ to Ge_136_ to Sn_136_, it is helpful to use a simple harmonic model in which the rattling frequencies are modeled as ω = (*K/M*)^1/2^. Here *M* is atomic mass of the guest and *K* represents an effective force constant characterizing the weak guest atom-host atom bonding. From the values of the LDA derived guest frequencies just mentioned, we can estimate the effective force constants *K* for the Na guests in the three materials. Using this procedure, we find that the effective force constant for Na in the Si_28_ cages is 0.44 eVÅ^−2^, that for Na in the Ge_28_ cages is 0.59 eVÅ^−2^, and that for Na in the Sn_28_ cages is 0.64 eVÅ^−2^.

These effective force constant results should be compared with a previous investigation of the effective force constant trends for Cs inside the large cages in Na_16_Cs_8_Si_136_, Na_16_Cs_8_Ge_136_, and Cs_24_Sn_136_ [20]. This previous study found that the effective force constant for Cs in the Sn_28_ cages is considerably reduced from both that for Cs in the Si_28_ cages and in the Ge_28_ cages. This Ref. [20] result was attributed to the large volume difference between the Cs atoms and the Sn_28_ cages, which causes the Cs guests to be very weakly bound to the cage framework. Similarly, the results just discussed for the Na guests in the Si_28_, Ge_28_, and Sn_28_ cages can be qualitatively understood by considering the size difference between the Na guest atom and the M_28_ cages. Specifically, the small Na atom size inside the large cages leads to the Na guests being very weakly bound, as indicated by the calculated small effective force constants *K*. The fact that the effective force constant for Na in the Si_28_ cages is slightly smaller than that for Na in either the Ge_28_ cages or in the Sn_28_ cage is not understood in detail. However, we speculate that this may be caused by a breakdown in the harmonic approximation for the Na guest atom modes. That is, an accurate treatment of the Na guest atom modes in the large cages in the Type II clathrates may require the inclusion of anharmonic terms in the effective Na-host material potential. The treatment of such anharmonic effects is beyond the scope of this paper.

Starting with the acoustic vibrational modes obtained in the LDA calculations just discussed, and taking the long wavelength limit (*k* → 0), the material elastic constants C_11_ and C_44_ and the corresponding sound velocities (v = ω/k) can be estimated. Here k and ω are the wave vector and the acoustic phonon frequency near the Γ point in the Brillouin Zone. Table 2 summarizes, for the six Type II clathrate based materials listed there, our calculated results for the elastic constants C_11_ and C_44_ and for the transverse and longitudinal acoustic phonon velocities (*v*_t_^[100]^ and *v_l_*^[111]^) along the [100] (Γ → X) and [111] (Γ → L) directions in the Brillouin zone. In the two right most columns of Table 2, our results for the above calculated effective force constants *K* and for the vibrational frequencies ω of the Na guests are also listed. As is clear from the elastic constants listed in Table 2, our results predict that the Ge clathrate compounds should be softer than the Si compounds and that the Sn compounds should be significantly softer than the Ge compounds. Also, the sound velocities in these materials follow a similar trend. That is, these velocities become smaller as the host material changes from Si_136_ to Ge_136_ to Sn_136_. The fact that the Sn based materials are predicted to have relatively small sound velocities is a strong indication that the lattice thermal conductivity in these materials should also be relatively small and thus that the thermoelectric figure of merit for these materials should be enhanced.

## 4. Conclusions 

In this paper, we have reported the results of a density functional based, first-principles study of the structural and vibrational properties of the Type II clathrate compounds A_x_Si_136_, A_x_Ge_136_, and A_x_Si_136_ for various x and for the guest atoms A = Na, K, Rb, and Cs. We have presented and discussed the results of our calculations for the variation of the lattice constants, the bulk moduli, and other structural parameters with guest atom composition x, and we have compared and contrasted these results for the three different Type II clathrate host materials. We have also presented and discussed the results of our calculations of phonon dispersion relations for Na_4_Si_136_, Na_4_Ge_136_, and Na_4_Sn_136_. We have also made qualitative and quantitative comparisons of the vibrational spectra for the three materials. Finally, we have presented the results of our calculations of the low frequency elastic constants and the longitudinal and transverse sound velocities in Na_x_Si_136_, Na_x_Ge_136_, and Na_x_Sn_136_ for *x* = 4 and 8 and we have compared and contrasted these results for these three different clathrate hosts.

## Figures and Tables

**Table 1:** LDA-derived Structural Parameters for the Materials Listed. Obtained from the Birch-Murnaghan Equation of State (EOS) (at *T* = 0 K) as Outlined in the Text.

Clathrate	E_0_ (eV/atom)	V_0_ (Å^3^/atom)	a_0_ (Å)	B (GPa)	dB/dP
Na_8_Si_136_	−5.65	21.43	14.558	83.70	3.84
K_8_Si_136_	−5.68	21.45	14.564	83.60	3.90
Rb_8_Si_136_	−5.68	21.49	14.572	82.99	4.77
Cs_8_Si_136_	−5.69	21.53	14.582	83.28	5.28
Na_8_Ge_136_	−4.94	24.46	15.216	58.84	4.51
Na_8_Sn_136_	−4.27	36.78	17.431	37.22	4.71

**Table 2:** Elastic Properties of Na_x_M_136_ (*x* = 4, 8; M = Si, Ge, Sn).

Clathrate	C_44_ (GPa)	C_11_ (GPa)	*v*_t_^[100]^ (m/s)	*v_l_*^[111]^ (m/s)	*K* (eVÅ^−2^)	ω (meV)
Na_4_Si_136_	26.60	92.35	3558	6175	0.44	6.2
Na_4_Ge_136_	20.02	75.85	2063	3848	0.59	7.2
Na_4_Sn_136_	9.77	50.9	1384	2929	0.64	7.9
Na_8_Si_136_	22.81	82.98	3253	6184	0.22	4.4
Na_8_Ge_136_	21.29	69.47	2118	3776	0.49	6.6
Na_8_Sn_136_	9.35	42.27	1351	2868	0.64	7.9
